# Let's not throw the baby out with the bath water: next steps for child obesity prevention in early life

**DOI:** 10.3389/fpubh.2026.1801182

**Published:** 2026-08-14

**Authors:** Rachel Laws, Konsita Kuswara, Dimity Dutch, Eve House, Ioanna Katiforis, Jessica Appleton, Penelope Love, Elizabeth Denney-Wilson

**Affiliations:** 1 Institute for Physical Activity and Nutrition, School of Exercise and Nutrition Sciences, Deakin University, Geelong, VIC, Australia; 2 NHMRC Centre of Research Excellence in Translating Early Promotion of Optimal Child Growth (CRE EPOCH-Translate), Sydney, NSW, Australia; 3 Sydney School of Public Health, Faculty of Medicine and Health, The University of Sydney, Sydney, NSW, Australia; 4 School of Public Health, University College Cork, Cork, Ireland; 5 Caring Futures Institute, College of Health and Enablement, Flinders University, Adelaide, SA, Australia; 6 Health Promotion Unit, Population Health Research & Evaluation Hub, Sydney Local Health District, Sydney, NSW, Australia; 7 School of Nursing and Midwifery, Faculty of Health, The University of Technology, Sydney, NSW, Australia; 8 Susan Wakil School of Nursing and Midwifery, The University of Sydney, Sydney, NSW, Australia

**Keywords:** child health & development, child obesity prevention, early life, health behaviors, primary health care

## 1 Introduction

The first 5 years of life is a critical stage for establishing optimal health behaviors to support early childhood growth, development and prevent early childhood overweight and obesity (1, 2). Health behaviors including dietary intake, physical activity, sedentary behavior, and sleep, established in childhood, are likely to track into adolescence and adulthood, influencing lifelong health and risk of chronic disease (3). The prevention of overweight and obesity in childhood remains a significant public health priority, with over 37 million children under 5 years living with overweight and obesity worldwide (4). Early growth patterns, particularly rapid weight gain during infancy, are strongly associated with increased risk of obesity (5) and cardiometabolic disorders in later life (6) as well as other adverse consequences including poorer health related quality of life which worsens with age (7), and poorer educational (8), social and emotional outcomes (1, 9). The World Health Organization (WHO) report of the Commission on Ending Childhood Obesity (10) recommends a range of strategies including ‘*provide guidance on, and support for healthy diet, sleep and physical activity in early childhood to ensure children grow appropriately and develop healthy habits'*. Given the central role of early childhood health behaviors in supporting optimal growth, health, and development, and shaping later obesity risk, it is essential to examine how well current parent-focused interventions influence these outcomes.

The Transforming Obesity Prevention for CHILDren (TOPCHILD) Collaboration is the largest international collaboration of planned, ongoing, and completed early childhood obesity prevention trials. Recent findings from the landmark TOPCHILD study examined individual participant data (IPD) from 31 parent-focused interventions (11). When data were examined from 17 trials reporting on the impact of these interventions on child Body Mass Index (BMI) z-score at 24-months of age, no evidence was found that the included interventions had an impact on BMI z-score (11). Furthermore, there was limited evidence of intervention effectiveness in changing child health behaviors, including diet, movement, and sleep, in the first 2 years of life (11). This may prompt re-consideration of the value of parent-focused obesity prevention interventions in early childhood.

## 2 Recommendations for practice: combining upstream policy and downstream family support

Like all complex public health problems, individual programs alone are not the solution, rather, coordinated, multi-level, multi-sectoral action embedded within existing systems is paramount (12). Broader policies addressing upstream social, economic, environmental, and commercial determinants of health are essential to improving the environments in which children live and grow. We argue however, that these upstream changes must be accompanied by downstream individual-level support for families to foster optimal health behaviors during the critical first 2,000 days of life.

Pregnancy and early childhood represent a unique window where families actively seek information and support on healthy growth and development (13) and contact with the primary health care (PHC) system is high (14). However, caregivers often face conflicting advice from family, friends, social media, and health professionals alike (15), while also navigating other pressing challenges such as financial hardship, food insecurity, insecure housing and mental health concerns that may affect their capacity to engage with health promotion messages (16, 17). Rather than competing with families' immediate priorities, support for health behaviors should be positioned as part of a broader approach to promoting child and family wellbeing, starting where families are at and responding to their individual needs and circumstances, using a ‘whole of child' and ‘whole of family' approach (18). This requires interventions that are not ‘stand-alone' but integrated with existing services and settings, tailored to the needs of diverse families, and supported by structural (e.g. improved neighborhood environment) and policy components. We therefore recommend the following actions to strengthen ‘downstream' support for families.

### 2.1 Build capacity for consistent evidence-based health behavior support in universal services

Almost half of all Australian children aged 0–5 years attend early childhood education and care (ECEC) services, spending more than 26 h per week in care (19). Participation in ECEC services is similarly high across other high-income countries, with average enrolment rates of around 85% (20). Extensive policy, systems, and environmental work in ECEC, exemplified by evidence-based programs such as Go NAPSACC (Go Nutrition and Physical Activity Self-Assessment for Child Care) (21), has demonstrated the capacity of these settings to implement structured, evidence-informed health promotion at scale. There is evidence that these approaches are effective, contributing to improvements in nutrition environments, physical activity practices, and service-level health promotion (22) as well as improving diet and reducing obesity in at risk populations (23).

During this period of life, children also have frequent contact with primary health care (PHC) professionals, particularly in general practice and child and family health settings (14, 24). Professionals in these settings are well-placed to provide health promotion and preventive care through regular and scheduled contacts such as routine health checks and immunization visits (25, 26). Provision of consistent and reinforcing health promotion messages from PHC and ECEC professionals may be more effective than isolated single-setting programs delivered at one point in time. Health behavior focussed messages may also overcome well known barriers and consequences of weight-focussed approaches including stigma and impact on rapport (27, 28). However, PHC professionals report gaps in their knowledge and skills needed to successfully embed early childhood health promotion into their practice, as well as a lack of funding and supporting resources to provide to families (29–32). Similarly, ECEC professionals have a profound influence on children in their care and are expected to support the development of healthy behaviors, however, the structures and training needed to implement health promotion effectively are often lacking (22). For example, Australian ECEC professionals and cooks receive limited formal training in providing healthy meals and encouraging healthy eating behavior, frequently relying on social media sources or food company brochures for nutrition information (33).

Thus, while policy, systems, and environmental initiatives have strengthened the broader health-promoting environment, there remains a need for practical tools, resources, and training to enable PHC and ECEC professionals to routinely monitor and promote child health behaviors (34, 35). There is ample opportunity to embed these tools and resources within guidelines and existing systems, including digital and physical child health records, the preschool health check, and electronic medical records, to enable consistent health promotion support and monitoring of child health behaviors across services and sectors (36).

There is also an important opportunity for better cross sector integration in programs and services that reach young children both within and outside of the health sector. For example, previous research with the Special Supplemental Program for Women, Infants and Children (WIC) Program and Pediatricians has found that families, providers and public health professionals are supportive of shared health assessment data between public health and clinical settings to support consistency in messaging to families (37). Further research is needed however to examine both the process for integrating care and the impact on child health outcomes.

### 2.2 Target support within and outside the health system to reach priority populations and diverse caregivers

Among the completed trials included in the TOPCHILD IPD (11), more than half (54%) targeted priority populations, including specific ethnic or racial groups and families facing psychological, social, and economic disadvantage. However, only a third of trials were successful in enrolling participants from these groups and less than a quarter involved priority populations in the design of the interventions (38). This highlights the importance of moving beyond trials delivered by researchers to engaging priority populations in intervention development and embedding programs into existing services and settings to reach families from diverse cultural and caregiving contexts. Such approaches may include co-designing programs with communities from the outset or adapting existing evidence-based interventions and implementation strategies to reflect family needs and service delivery contexts.

The importance of developing culturally responsive interventions is especially relevant to culturally diverse countries such as Australia and the United States and when evidenced based interventions developed within Western cultural and knowledge systems are adapted for populations with different cultural worldviews, including Indigenous peoples and other non-Western communities. Existing evidence suggests that cultural adaption of early childhood obesity interventions can improve acceptability among target cultural groups (39). However, the impact on program reach and child health behaviors are less well understood and cultural adaption processes are generally poorly described (39). Although several frameworks exist to guide cultural adaptions of health interventions (40, 41), they generally give limited consideration of how interventions are scaled and delivered in real world settings. Implementation science frameworks may help address this gap by incorporating equity and contextual considerations into adaption processes. For example, the Practical, Robust Implementation and Sustainability Model (PRISM)(42) considers the dynamic interactions between an evidence-based intervention, the perspective of diverse recipients, the implementation infrastructure supporting program delivery, and the external environment (e.g. broader policies, funding structures), providing a useful lens for adapting and scaling interventions for diverse populations (43).

It is also important to conceptualize ‘parents' as encompassing not only mothers, but also fathers, same sex partners, grandparents, and broader family members. Our own research (44) and that of others (45) has shown that fathers perceive that they have an important role to play in promoting positive nutrition and physical activity behaviors in their children, however father-specific information and support are often lacking and there are very few pediatric obesity prevention trials that have involved fathers (46). Similarly, very few studies have investigated the influence of grandparents on child nutrition and physical activity behaviors or engaged grandparents in intervention programs (47). Research with parents who identify as LGBTQI+ is limited, with existing studies suggesting the need for tailored support for feeding (48) and additional training for health professional to ensure respectful care during the perinatal period (49). Collectively, these gaps highlight the need for more inclusive programs that recognize and actively engage the diversity of caregivers involved in shaping children's nutrition and physical activity behaviors.

## 3 Recommendations for future research: improving design, measurement, and scale-up of parent-focused interventions

The findings from the TOPCHILD IPD (11) highlight the importance of considering how we evolve the design, conduct, and evaluation of parent-focused interventions to improve their effectiveness, fully capture their impact, and consider how they can be scaled, implemented, and sustained in routine service delivery.

### 3.1 Identify effective intervention components

Developing the next generation of effective early childhood health promotion and obesity prevention interventions requires a clear understanding of what makes interventions effective. While the TOPCHILD IPD found no overall effect of interventions on BMI z-score from across 17 trials (11), some of these interventions reported positive intervention effects when considered as individual programs. Therefore, an exploration of effective intervention components (an analysis planned by TOPCHILD investigators) (50), as well as a holistic examination of the characteristics of effective programs is needed, given that components within programs are likely to work synergistically. Future analyses should also examine whether particular intervention components are more effective for specific groups of children or families to inform the development of more tailored and responsive interventions. Further research is also needed to identify delivery modes to maximize reach and engagement. In this context, digital health approaches offer considerable potential to deliver personalized interventions at scale, although further work is required to evaluate both engagement with, and the effectiveness of these interventions in improving child health behaviors during the first 2,000 days of life (51).

### 3.2 Standardize outcome measures beyond zBMI

Designed for population-level comparison, rather than individual monitoring, there are well-documented statistical limitations of using BMI-z scores for detecting changes in weight or adiposity over time (52). These limitations are further amplified when BMI z-scores are used to inform health promotion advice to families, as growth charts are often completed or interpreted inaccurately, inconsistently, or inappropriately (53). Collectively, these limitations underscore the need for research and practice to prioritize measuring behavioral outcomes, such as diet, physical activity, sedentary behavior, and sleep as more appropriate targets of intervention.

However, findings from the TOPCHILD IPD demonstrate the significant variability in how early childhood health behavior outcomes are currently measured and reported in trials (11). Therefore, we also advocate for the use of Core Outcome Sets (COS) and Core Outcome Measurement Sets (COMS) to standardize recommendations on what and how outcomes are measured. COS and COMS aim to overcome measurement heterogeneity, support evidence synthesis, and reduce research waste (54, 55). COS and COMS for early childhood health behaviors are currently being developed, and aim to support standardized measurement across research, policy, and practice (56–58). Recommendations to standardize the measurement of sociodemographic data, including indicators of socio-economic position and ethnicity/race, will further strengthen evidence synthesis (38). To ensure we capture the full benefit of health promotion and obesity prevention programs, it is important that trials also measure the concurrent benefits of programs (i.e. ‘spillover' effects) on parents and caregivers shown in some trials (59). Additionally, co-benefits for children beyond obesity risk and health behaviors, such as impacts on child development, wellbeing, school readiness, and educational outcomes should be included (56).

### 3.3 Test approaches to scale up and embed effective programs in policy and practice

Very few early life obesity prevention programs have been scaled up (60), with little known about the most effective implementation strategies to scale programs, achieve equitable reach to families most in need, and embed programs into existing systems for long-term sustainability. Engaging established implementation science frameworks, such as the Consolidated Framework for Implementation Research (CFIR) (61), can inform planning and decision-making around scaling programs by providing structured approaches to assess barriers, facilitators and contextual factors influencing implementation, and guide the development of tailored strategies to support sustained implementation at scale. Evidence of a program's effectiveness alone is insufficient for integration into routine practice (62).

Successfully embedding programs into routine practice requires sustained implementation at local and organizational levels, with ongoing investment, effective governance and partnerships, and supportive systems (62). Adapting programs and implementation strategies to fit local needs and context, ensuring adequate funding, securing buy-in from stakeholders, and aligning with government priorities are key factors for successful scale-up (63). Real-world examples demonstrate the potential of leveraging established delivery infrastructure, and community trust to support equitable and sustained implementation at scale. Examples include behavior change interventions embedded within existing delivery systems, such as the Early Intervention to Promote Cardiovascular Health of Mothers and Children (ENRICH) intervention (64), delivered within an evidence-based home visiting program, and the INfant Feeding, Active play and NuTrition (INFANT) program (65), delivered in partnership with universal maternal and child health services.

### 3.4 Evaluate policy and structural components within behavior change interventions

Much of the existing research has focused on examining the effectiveness of health behavior change interventions for early childhood obesity prevention in isolation from policy level approaches. Consistent with recommendations from the European EndObesity Consortium (66), there is an opportunity to more robustly assess the combined effects of policy level or structural components alongside child health behavior change interventions. Structural components may include incentives to improve access to healthy foods such as food vouchers, improving service availability and access and enhancing aspects of the neighborhood environment. An example of this integrated evaluation is The ECAIL study in France which is examining the effectiveness of combining home visits by a dietitian with financial incentives that reduce the cost of kitchen utensils and healthy foods for disadvantaged families (67).

## 4 Conclusion

Early childhood health promotion and obesity prevention remains a complex and critical public health priority. While recent evidence calls into question the effectiveness of parent-focused interventions tested in research trials, this does not diminish the need to continue to provide guidance to families to support optimal health behaviors in early childhood. We argue that this requires a comprehensive approach that combines upstream policy and environmental systems change with downstream family support. Such support should be positioned within a broader ‘whole of child' and ‘whole of family' approach to wellbeing, with interventions integrated into existing services and settings, tailored to the needs of diverse families, and supported by complementary structural and policy actions. Parent-focused programs are essential but not sufficient on their own, underscoring the multiple interacting factors that influence healthy growth and development in early childhood, many of which are entirely outside the influence of parents and caregivers. Rather than abandoning parent-focused programs, we must support their evolution and advocate for accompanying system-level changes to create healthy environments for our future generations to thrive.
